# Cortex Fraxini (Qingpi) Protects Rat Pheochromocytoma Cells against 6-Hydroxydopamine-Induced Apoptosis

**DOI:** 10.1155/2015/532849

**Published:** 2015-08-10

**Authors:** Jing-Jie Li, Shi-Ya Zhou, Huan Zhang, Kim-Hung Lam, Simon Ming-Yuen Lee, Peter Hoi-Fu Yu, Shun-Wan Chan

**Affiliations:** ^1^Department of Applied Biology and Chemical Technology, The Hong Kong Polytechnic University, Hong Kong; ^2^School of Biological Sciences, The University of Hong Kong, Hong Kong; ^3^Food Safety and Technology Research Centre, Department of Applied Biology and Chemical Technology, The Hong Kong Polytechnic University, Hong Kong; ^4^State Key Laboratory of Quality Research in Chinese Medicine and Institute of Chinese Medical Sciences, University of Macau, Avenue Padre Tomás Pereira S.J., Taipa, Macau; ^5^Faculty of Science and Technology, Technological and Higher Education Institute of Hong Kong, Hong Kong; ^6^State Key Laboratory of Chinese Medicine and Molecular Pharmacology, Shenzhen, China

## Abstract

Parkinson's disease (PD) is a chronic neurodegenerative disorder having close relationship with oxidative stress induced by reactive oxygen species (ROS). Cortex Fraxini (QP) is a kind of traditional Chinese medicinal herb with antioxidant properties. It may be a potential candidate for preventing the development of chronic neurodegenerative diseases. Thus, the key objective of the current study was to investigate the neuroprotective effect of QP water extract on 6-hydroxydopamine (6-OHDA) induced apoptosis in rat pheochromocytoma (PC12) cells. It was found that QP water extract possesses strong antioxidant property with SC_50_ = 0.15 mg/mL. Total phenolic content of QP water extract was found to be 200.78 ± 2.65 mg GAE/g. QP water extract's free radical scavenging capacity was demonstrated by reversing the increased level of intracellular ROS induced by 6-OHDA, using 2′,7′-dichlorodihydrofluorescein diacetate. Moreover, QP water extract (0.5 mg/mL) could remarkably increase the viability of PC12 cells treated with 6-OHDA. The protective effect of QP water extract was found to be via inhibiting MEK/ERK pathway and reversing PI3-K/Akt/GSK3*β* pathway. The current results suggest that QP might be a potential candidate for preventing the development of neurodegenerative diseases, such as PD.

## 1. Introduction

Parkinson's disease (PD), which frequently occurs among elderly especially those beyond 65, is a chronic neurological disorder with some typical characteristic symptoms such as tremor, myotonia, and dyskinesia [1]. The pathophysiology of PD has not been thoroughly understood except some evidence showing that PD may be partly caused by nigral cell death [2].

6-Hydroxydopamine (6-OHDA), a neurotoxin, is mainly exerted neurotoxicity through two pathways including producing free radicals or blocking the mitochondrial respiratory chain [3]. Both pathways would lead to the formation of reactive species of oxygen (ROS) and increase of oxidative stress, which would interact with intercellular molecules (DNA, proteins, and lipid) and finally lead to neurotoxicity, including the loss of dopaminergic neurons in the substantia of nigra [4]. 6-OHDA can be transported into neurons by plasma membrane dopamine transporters and inhibits the mitochondrial electron transport chain complexes I and IV, resulting in ROS-induced neuronal damage as observed in PD [5, 6]. Therefore, 6-OHDA is thought to induce toxicity, that is similar to the biochemical and neuropathological conditions in PD, and commonly used either* in vitro* or* in vivo* to mimic PD [6].

Cotex Fraxini (QP), a traditional Chinese herb, is the dried bark of* Fraxinus rhynchophylla *Hance,* Fraxinus chinensis *Roxb.,* Fraxinus chinensis *Roxb. var*. acuminate *Lingelsh., and* Fraxinus stylosa *Lingelsh. usually from Jilin, Liaoning, and Hebei in China. It is a well-known herb belonging to the “heat-clearing” category in the classification of traditional Chinese medicine (TCM) [7] and frequently utilized to cure diarrhea, bacillary dysentery, arthritis, and hyperuricemia [8]. QP was found to possess abundant antioxidant constituents including phenolic acid and coumarins [9]. Therefore, it may have the potential to protect neuronal cells from ROS damage and prevent the development of neurodegenerative diseases. As a result, the aims of the current study were to investigate the antioxidant property of QP water extract and to evaluate the neuroprotective effect of QP water extract in 6-OHDA-induced apoptosis in rat pheochromocytoma (PC12) cells. This* in vitro *model could mimic the oxidative stress induced cell death in PD [10]. Further mechanistic study was performed to unveil the antiapoptotic role of QP water extract in 6-OHDA-induced apoptosis in PC12 cells.

## 2. Materials and Methods

### 2.1. Chemicals and Reagents

Dulbecco's modified Eagle's medium (DMEM), fetal bovine serum (FBS), phosphate buffer saline (PBS), Horse serum (HS), antibiotics and antimycotics, trypsin 3-(4,5-dimethylthiazol-2-yl)-2,5-diphenyltetrazolium bromide (MTT) and Hoechst 33342 were purchased from Invitrogen (Carlsbad, CA, USA). PD98059, SB415286, and LY294002 were obtained from LC laboratories (Woburn, MA, USA). ECL plus kit were purchased from Amersham Bioscience (Aylesbury, UK). 2′, 7′-Dichlorodihydrofluorescein diacetate (DCFH-DA), ethyl alcohol (absolute ethanol), and dimethyl sulfoxide (DMSO) were purchased from Sigma-Aldrich Inc. (St. Louis, MO, USA). Cell lysis buffer, antibodies against phospho-MEK 1/2, phospho-ERK 1/2, ERK, phospho-PI3K, phospho-Akt, Akt, phospho-GSK3*β*, GSK3*β* were obtained from Cell Signaling Technology (Beverly, MA, USA). Antibody against *β*-actin was obtained from Santa Cruz Biotechnology (Santa Cruz, CA, USA). All other chemicals used were of analytical grade.

### 2.2. Medicinal Herb Extract

QP was purchased from Chinese pharmacy in Hong Kong, China. 50 g of QP was weighed and put into 300 mL boiling distilled water for 1.5 h to extract its water-soluble substances. Given that the residues still contain part of substance which cannot be extracted in one time, the reextraction was performed with 300 mL boiling ultrafiltered water for another two times. QP water extract was then centrifuged at 5,000 rpm for 15 min to obtain a clear supernatant for freeze-drying at −40°C for 48–72 h to acquire dry powder. The freeze-dried powder was kept at −20°C before use. QP freeze-dried powder (QP water extract) was redissolved in water or culture medium to prepare solutions with various concentrations for different experiments.

### 2.3. High-Performance Liquid Chromatography (HPLC) Analysis on QP Water Extract

HPLC analysis was performed using an Agilent 1100 HPLC system (Milford, MA, USA) coupled to a photodiode array detector. The samples were separated on a Supelcosil LC-18 column (250 mm × 4.6 mm; 5 *μ*m) (Supelco, Bellefonte, PA, USA) and eluted at a flow rate of 1 mL/min at 25°C. The solvent system consisted of methanol-water-glacial acetic acid (30 : 70 : 4, v/v/v), and the detection wavelength was set at 348 nm. QP water extract sample was dissolved in distilled water and filtered by a 0.45 *μ*m filter before HPLC analysis. The injection volume was 5 *μ*L.

### 2.4. Cell Culture and Treatment

The PC12 cells were obtained from ATCC (Manassa, VA, USA) and cultured in DMEM supplemented with 10% FBS, 1% HS, penicillin (100 U/mL), and streptomycin (100 *μ*g/mL) in a humidified atmosphere of 95% air and 5% CO_2_ at 37°C. For the experiments with 6-OHDA, PC12 cells in DMEM with low serum content (2% FBS and 1% HS) were seed in 6-well plate or 96-well plate. PC12 cells were pretreated with QP water extract at various concentrations for 2 h, before exposure to 6-OHDA for 4 h.

### 2.5. Determination of Antioxidant Properties

In order to measure the antioxidant properties of QP water extract, DPPH free radical scavenging activity assay and total phenolic quantification are induced in this study. For DPPH assay, various concentrations of QP water extract and vitamin C were prepared by dissolving the dried powder with distilled water. 50 *μ*L of sample solution was added to 950 *μ*L of DPPH^•^ methanol solution (24 mg/L) and allowed to react for 1 h. The scavenging activity of each sample was measured by spectroscopic method. Absorbance of sample (*A*_sample_) was determined by UV-visible spectrometer (Perkin Elemer Lambda 35) at 515 nm. Using 50 *μ*L of water as control (*A*_control_), the free radical scavenging rate (SR%) of each concentration was calculated by the following equation:

(1) $$\begin{array}{c} SR\%=\left(1-\frac{A_{sample}}{A_{control}}\right)\times 100\%. \end{array}$$

$\;A_{control}$ is absorbance value of DPPH^•^ solution with 50 *μ*L of water.
 
*A*_sample_ is absorbance value of DPPH^•^ solution with 50 *μ*L of herbal extract.

The 50% scavenging concentration (SC_50_) was calculated by GraphPad Prism 5.01 for Windows (GraphPad Software, San Diego California, USA).

For the determination of total phenolic content of QP water extract, Folin-Ciocalteu method was used to measure the total phenolic quantification of QP water extract, following the procedures we described before [11]. 100 *μ*L of sample solutions (QP water extract (0.3, 0.5 and 1.0 mg/mL) and gallic acid (0.01, 0.02, 0.03, 0.04, 0.05, 0.06, 0.07, and 0.08 mg/mL)) were mixed with 400 *μ*L of Na_2_CO_3_ (75.05 g/L) and 500 *μ*L of Folin-Ciocalteu reagent (1 : 10 diluted with water). The mixtures were then placed at room temperature for 2 h to allow reaction to take place. Absorbance of samples at 750 nm was determined by UV-visible spectrometer (Perkin Elemer Lambda 35). A standard curve was prepared by using different concentrations of freshly prepared gallic acid solutions. Total phenolic contents of the herbs were expressed as gallic acid equivalents (mg GAE/g).

### 2.6. Measurement of Cell Viability

After treatment with different stimuli, the medium was removed and replaced with the medium (100 *μ*L/well), adding with MTT solution (5 mg/mL in PBS). After 4 h incubation at 37°C, the cell supernatants were discarded and 100 *μ*L DMSO was used to dissolve MTT crystals. The absorbance of the samples was then measured at a wavelength of 570 nm with 655 nm as a reference wavelength. All assays were performed in triplicate.

### 2.7. Determination of Intracellular ROS Level

To evaluate the production of intracellular ROS with the treatment of 6-OHDA and QP water extract, 2′,7′-dichlorodihydrofluorescein diacetate (DCFH-DA) is used in the study based on the fact that intracellular deacetylation and oxidation of DCFH-DA to 2′,7′-dichlorodihydrofluorescein (DCF), which is highly fluorescent [12].

PC12 cells were seeded in black 96-well plates at a density of 1.5 × 104 cells per well and grown at 37°C for 24 hours, and then DCFH-DA (20 *μ*M) was added for 1 h pretreatment. After changing the medium, the cells were pretreated with Qinpi (0.5 mg/mL) for 2 hours and then the medium was replaced with no phenol red DMEM before 500 *μ*M 6-OHDA being added. The florescent intensity of DCF was scanned with a plate reader (Wallac; PerkinElmer) at 485 nm excitation and 535 nm emission. The results were expressed as folds increase of control.

### 2.8. Nuclear Staining for Assessment of Apoptosis

Hoechst staining assay is used to detect the change in morphology after exposing to 6-OHDA. Hoechst 33342 is the most commonly used cell-permanent nuclear counter stain that emits blue fluorescence to distinguish condensed pyknotic nuclei in apoptotic cells. After pretreating PC12 cells with 0.5 mg/mL QP water extract for 2 h and incubation with 500 *μ*M 6-OHDA for 2 h in a 12-well plate (2 × 10^6^ cells/well), the medium was removed and all the wells were washed by ice-cold PBS. Cells were then stained by Hoechst 33342 (5 *μ*g/mL) for 5 min at 4°C. Nuclei could be observed using a fluorescence microscope at 400x magnification.

### 2.9. Flow Cytometry Analysis of Apoptosis

Apoptosis in PC12 cells was analyzed through the measurement of mitochondrial membrane potential quantification by flow cytometry using JC-1 dye (Molecular Probes). After incubation with 6-OHDA, CCCP (carbonyl cyanide m-chlorophenylhydrazone, positive control) and QP water extract, PC12 cells were collected and centrifuged at 1200 rpm for 5 min. Removing the supernatants, the cell pellets were washed twice and resuspended in 500 *μ*L cold PBS. Then the cell suspension was transferred to 1.5 mL microcentrifuge tubes and centrifuged at 1200 rpm for 3 min. Aspirating the supernatant, cell pellets were resuspended in 500 *μ*L warm PBS. Then, 4 mM JC-1 dye was added to each sample to make the final concentration as 15 *μ*M before incubation at 37°C for 20 min. The proportion of aggregated versus monomeric JC-1 probe was quantified using the ratio of fluorescence emissions at 590 nm (red) over 530 nm (green) with a FACSCalibur flow cytometer. Consequently, the mitochondrial depolarization, which means the loss in mitochondrial membrane potential, is indicated by a decrease in the red/green fluorescence intensity ratio.

### 2.10. Western Blot Assay

Briefly, cells were collected after incubation in 6-well plate, using cell lysis buffer with 1 mM PMSF. After incubation on ice for 10 min and centrifugation at 14,000 rpm at 4°C for 15 min, the whole protein concentrations were determined by the BCA assay (Pierce, Rockford, IL, USA) using bovine serum albumin as standard, after which cell protein was diluted in the SDS sample buffer, and the mixture was boiled for 5 min. The protein (30 *μ*g) was separated on a 10% SDS-polyacrylamide gel. Then, the signals were transferred onto a polyvinyl difluoride membrane. Using 5% milk for 1 h, the signals were blocked on the membrane and detected with primary antibodies. After incubation overnight at 4°C, signals were obtained by binding a secondary antibody. The membrane was visualized using an ECL plus kit before exposure to FujiFilm autoradiographic films according to the manufacturer's protocol.

### 2.11. Statistics Analysis

All data are expressed as means ± SEM. Differences in the mean among groups were assessed for significance by one-way ANOVA combined with the Bonferroni's test. Differences were considered significant when *P* < 0.05. All statistical analysis tests were performed by GraphPad Prism 5.01 for Windows.

## 3. Results

### 3.1. HPLC Analysis

**Figure 1:**
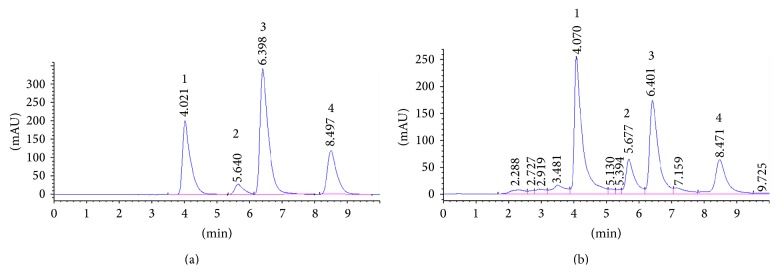
Representative HPLC-UV chromatograms of (a) mixed standards and (b) QP extract. Peaks: 1 is exculin hydrate, 2 is fraxin, 3 is esculetin, and 4 is flaxetin.

In order to ensure the quality consistency and standardization of the sample tested, the chemical characteristics of the QP water extract were determined using the HPLC-UV method. Representative HPLC chromatograms of mixed standards and QP extract were shown in Figure 1. The QP water extract (9.8 mg) was found to contain exculin hydrate (1639.7 ± 363.1 *μ*g), fraxin (1355.3 ± 197.4 *μ*g), esculetin (163.4 ± 11.8 *μ*g), and flaxetin (33.3 ± 6.0 *μ*g).

### 3.2. Determination of Antioxidant Properties

**Figure 2:**
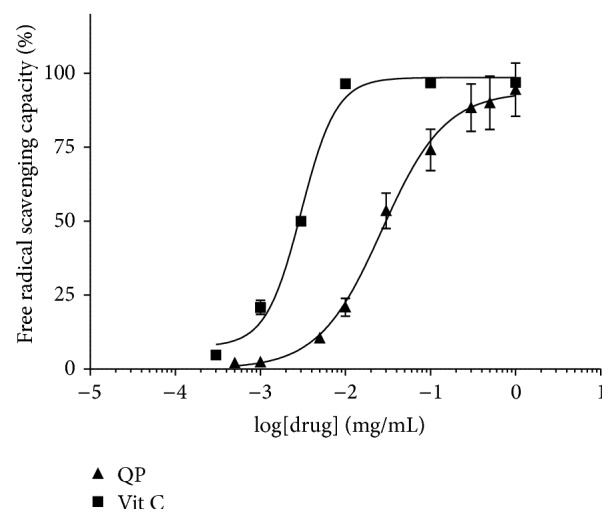
DPPH free radical scavenging capacity of QP water extract and Vitamin C (Vit C).

For DPPH assay, as is shown in Figure 2, QP water extract exerted a concentration-dependent scavenging activity against DPPH free radicals with SC_50_ of 0.15 mg/mL compared with that of ascorbic acid (Vitamin C, a positive control), which is 0.0030 mg/mL. In addition, the total phenolic content of QP water extract was found to 200.78 ± 2.65 mg GAE/g dry extract.

### 3.3. QP Water Extract Attenuates 6-OHDA-Induced Cell Death in PC12 Cells

**Figure 3:**
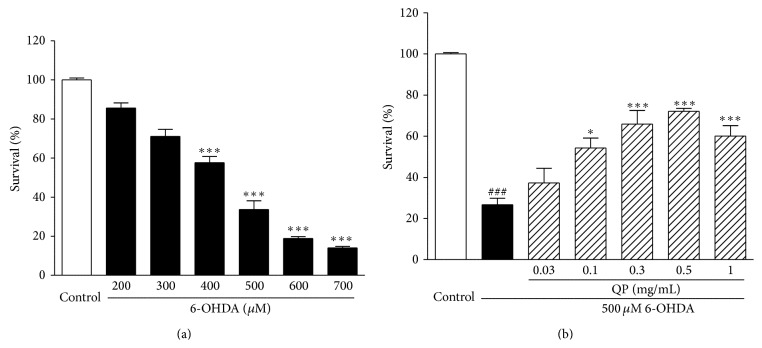
Examination of cytotoxicity of 6-OHDA and the effects of QP water extract on PC12 cells. (a) Cells were treated with different concentrations (200, 300, 400, 500, 600, or 700 *μ*M) of 6-OHDA for 6 h. Data were expressed as the means ± SEM of three separate experiments; _ _^*∗∗∗*^ *P* < 0.001 versus control. (b) PC12 cells were pretreated with QP water extract (0.03, 0.1, 0.3, 0.5, or 1 mg/mL, 2 h) before challenging with 6-OHDA (500 *μ*M, 6 h). Data were expressed as the means ± SEM of three separate experiments; _ _^###^ *P* < 0.001 versus control and _ _^*∗*^ *P* < 0.05 and _ _^*∗∗∗*^ *P* < 0.001 versus 6-OHDA alone group.

To analyze the cell injury induced by 6-OHDA, PC12 cells were exposed to 6-OHDA for 6 h with different concentrations ranging from 0 to 700 *μ*M. As shown in Figure 3(a), the viability of the cells treated with 6-OHDA of 400, 500, 600, and 700 *μ*M decreased significantly as compared with the control group (all with *P* < 0.001). The concentration of 6-OHDA (500 *μ*M) was selected for the subsequent experiment for inducing apoptosis in PC12 cells.

Compared to the cells treated with 6-OHDA (500 *μ*M) alone, 2 h pretreatment of QP water extract could concentration-dependently (0.03 mg/mL: 37.27 ± 7.10%, *P* > 0.05; 0.1 mg/mL: 54.30 ± 4.79%, *P* < 0.05; 0.3 mg/mL: 65.89 ± 6.56%, *P* < 0.001; 0.5 mg/mL: 72.10 ± 1.44%, *P* < 0.001; 1 mg/mL: 60.08 ± 5.04%, *P* < 0.001) improve cell viability in PC12 cells challenged with 6-OHDA (500 *μ*M) (Figure 3(b)). It was found that QP water extract (0.5 mg/mL) could provide the highest protection on the PC12 cells.

### 3.4. QP Water Extract Reduces the Level of Intracellular ROS in PC12 Cells Caused by 6-OHDA

**Figure 4:**
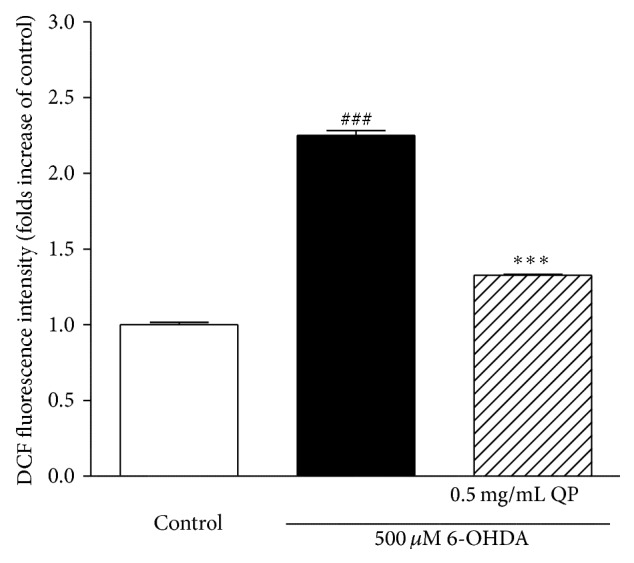
The effects of QP water extract on the changes of intracellular ROS caused by 6-OHDA. The PC12 cells were subjected to DCFH-DA (20 *μ*M) for 1 h followed by 2 h pretreatment with QP water extract (0.5 mg/mL), and then the PC12 cells were exposed to 500 *μ*M 6-OHDA for 2 h before detection of DCF fluorescent intensities. Data were expressed as fold increases of control with means ± SEM of three separate experiments; _ _^###^ *P* < 0.001 versus control and _ _^*∗∗∗*^ *P* < 0.01 versus 6-OHDA alone group.

As free radical scavenging capacity plays an important role in antioxidants which has the potential to protect the cells against oxidative stress-induced apoptosis, the measurement of intracellular ROS was used in this study to evaluate the effects of QP water extract on the changes of intracellular ROS level or the oxidative stress caused by 6-OHDA in PC12 cells. As shown in Figure 4, pretreatment with 0.5 mg/mL QP water extract could reverse the increase of intracellular ROS caused by 6-OHDA in PC12 cells to a certain level.

### 3.5. QP Water Extract Decreases the Apoptosis Induced by 6-OHDA in PC12 Cells

**Figure 5:**
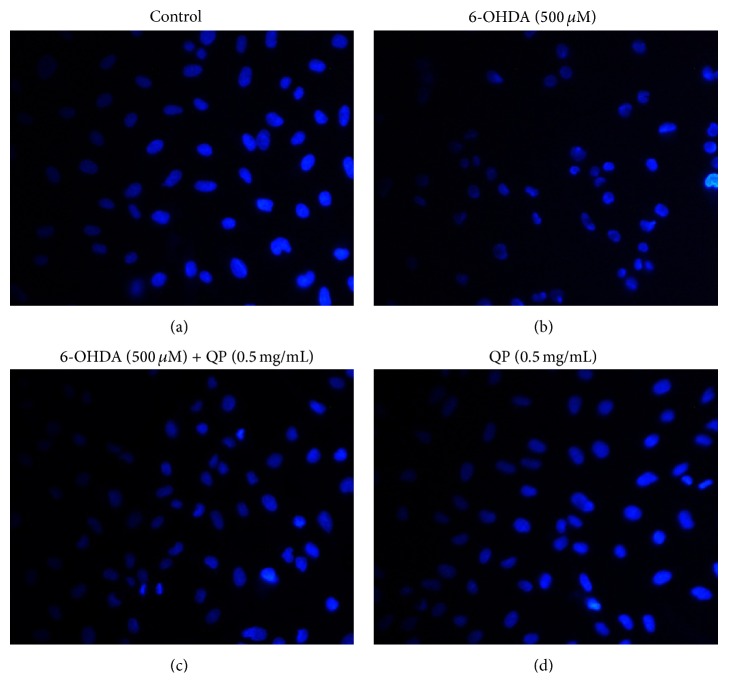
PC12 cells treated with different agents before Hoechst staining under the detection of 400x fluorescence microscope.

**Figure 6:**
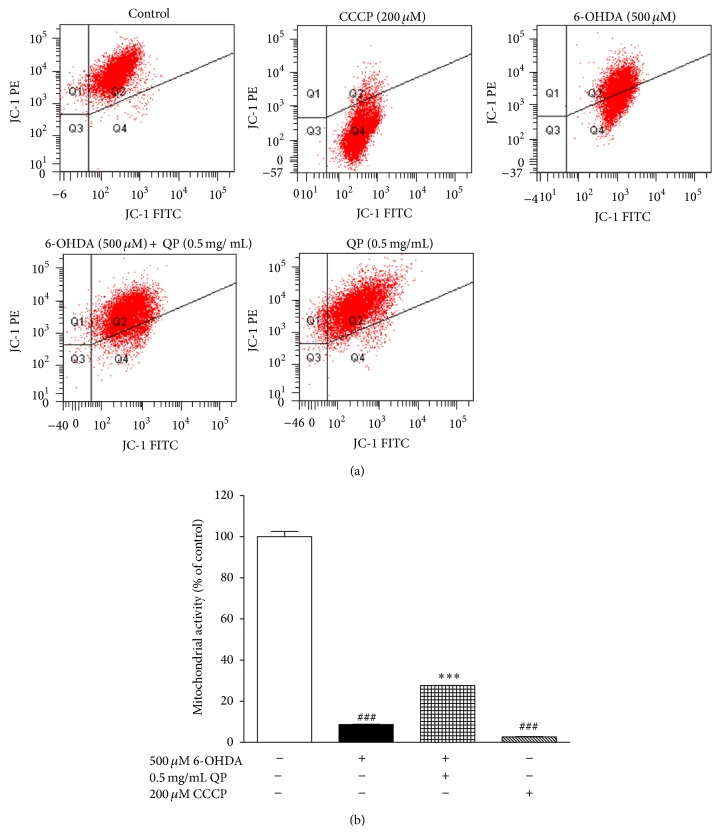
QP water extract attenuated 6-OHDA-induced apoptosis in PC12 cells. (a) Effects of QP water extract (0.5 mg/mL) on the changes of mitochondrial membrane potential induced by 6-OHDA (500 *μ*M). (b) Statistical analysis of the red/green fluorescence intensity ratio, namely mitochondrial membrane potential, expressed as the percentage of control. Data were expressed as means ± SEM of three separate experiments; _ _^###^ *P* < 0.001 versus control and _ _^*∗∗∗*^ *P* < 0.001 versus 6-OHDA alone group.

Nucleus staining assay with Hoechst 33342 was taken to evaluate the morphological changes, which indicated apoptosis in PC12 cells. After observing cells under the fluorescence microscope (×400 magnification), compared with the morphological character in control group, the group treated with 6-OHDA (500 *μ*M, 2 h) exhibited the symptom of apoptosis, including shrinkage of nuclei and condensation of chromatin appearance of a few apoptotic bodies and less number of cells (Figure 5). In contrast, cells pretreated with QP water extract (0.5 mg/mL, 2 h) showed less signs of apoptosis (Figure 5). Additionally, flow cytometry was used to further assess the apoptosis of PC12 cells through the measurement of mitochondrial membrane potential. As shown in Figure 6(a), pretreatment with QP water extract (0.5 mg/mL, 2 h) could reverse the PC12 mitochondrial membrane dysfunction induced by 6-OHDA (500 *μ*M, 4 h) (Figure 6(b)).

### 3.6. QP Water Extract Attenuates 6-OHDA-Induced Apoptosis in PC12 Cells by Inhibiting ERK Pathway

**Figure 7:**
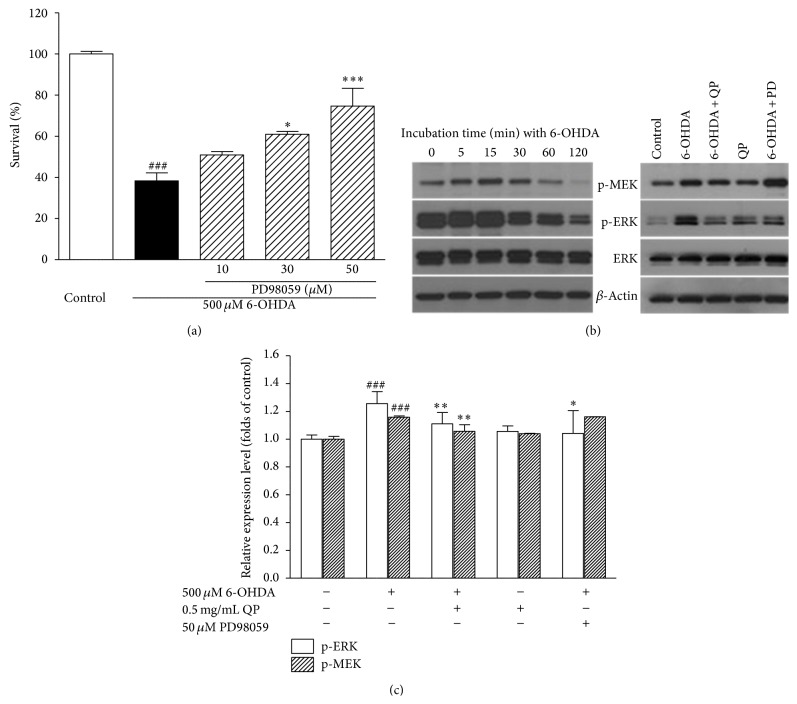
QP water extract exerted neuroprotective effect on 6-OHDA treated PC12 cells through inhibiting ERK pathway. (a) Pretreatment of PD98059 for 2 h prevented 6-OHDA-induced cell apoptosis in a concentration-dependent manner. Data were expressed as means ± SEM of three separate experiments, with _ _^###^ *P* < 0.001 versus control and _ _^*∗∗∗*^ *P* < 0.001 and _ _^*∗*^ *P* < 0.05 versus 6-OHDA alone group. (b) The increase of phospho-ERK and phospho-MEK caused by 6-OHDA (500 *μ*M) at 15 min was reversed by pretreatment with QP water extract (0.5 mg/mL, 2 h). (c) The band from three independent experiments were quantified by densitometry and shown as group. Calculated as fold of control, data were expressed as means ± SEM of three separate experiments, _ _^###^ *P* < 0.001 versus control and _ _^*∗∗*^ *P* < 0.01 and _ _^*∗∗*^ *P* < 0.05 versus 6-OHDA alone group.

To determine whether the ERK (extracellular signal-regulated kinases) pathway is involved in 6-OHDA induced apoptosis in PC12 cells, PD98059, a specific inhibitor of MEK, was used to pretreat PC12 cells for 2 h before exposure to 6-OHDA. 6-OHDA-induced cell apoptosis was the most significantly suppressed by 50 *μ*M PD 98059 (74.65 ± 8.61%, *P* < 0.001), compared with those treated with 500 *μ*M 6-OHDA alone (Figure 7(a)).

In addition, to prove whether 6-OHDA-induced apoptosis in PC12 cell is reversed by QP water extract through inhibiting the phosphorylation of ERK pathway, the levels of phospho-MEK and phospho-ERK are determined using Western blot assay. As shown in Figure 7(b), the level of phospho-ERK increased sharply to a peak 15 min after exposure to 500 *μ*M 6-OHDA, therefore, we selected to collect the protein at 15 min. It can be seen from the Figures 7(b) and 7(c) that pretreatment with QP water extract (0.5 mg/mL, 2 h) could significantly prevent the increase of phospho-MEK and phospho-ERK in PC12 cells, resulting from oxidative stress caused by 6-OHDA.

### 3.7. QP Water Extract Reverses the 6-OHDA-Induced Suppression of PI3-K/Akt/Gsk3*β* Pathway in PC12 Cells

**Figure 8:**
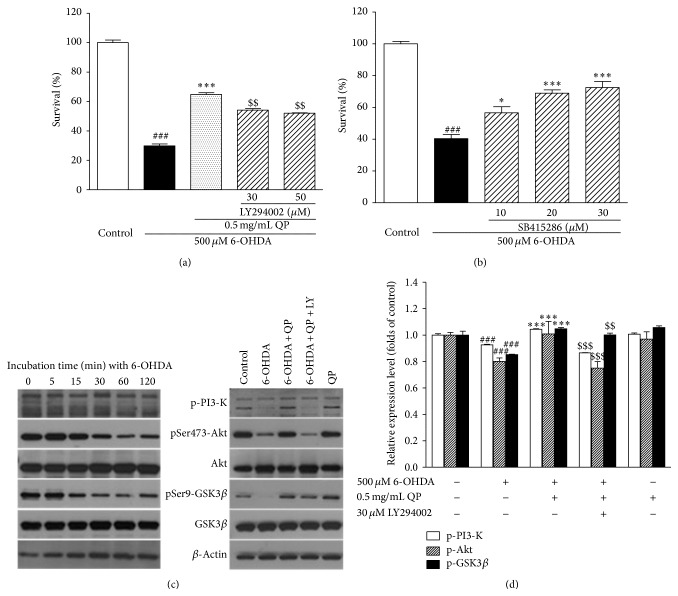
QP water extract exerted neuroprotective effect on 6-OHDA treated PC12 cells through activating PI3-K/Akt/GSK3*β* pathway. ((a), (b)) The neuroprotective effect of QP water extract on PC12 cells against 6-OHDA can be reversed by LY294002 and in a concentration-dependent manner. PC12 cells were exposed to 500 *μ*M 6-OHDA at 2 h after pretreatment with both QP water extract (0.5 mg/mL) and LY294002 and SB415286 at different concentrations as indicated. Data, expressed as percentage of control, were expressed as means ± SEM of three separate experiments; _ _^###^ *P* < 0.001 versus control, _ _^*∗∗∗*^ *P* < 0.01 and _ _^*∗*^ *P* < 0.05 versus 6-OHDA alone group, and _ _^$$^ *P* < 0.01 versus 6-OHDA with pretreatment of QP water extract group. (c) QP water extract had reversed the decline of phospho-PI3-K, phospho-AKT, and phospho-GSK3*β* caused by 6-OHDA to a certain level _ _^$$$^ *P* < 0.001 and _ _^$$^ *P* < 0.01 versus 6-OHDA with QP water extract pretreatment group. PC12 cells were pretreated with QP water extract (0.5 mg/mL, 2 h) and then exposed to 6-OHDA (500 *μ*M) for 60 min. (d) The bands from three independent experiments were quantified by densitometry and shown as group. Calculated as fold of control, data were expressed as means ± SEM of three separate experiments; _ _^###^ *P* < 0.001 versus control, _ _^*∗∗∗*^ *P* < 0.001 versus 6-OHDA alone group, and _ _^$$$^ *P* < 0.001 and _ _^$$^ *P* < 0.01 versus 6-OHDA with QP water extract pretreatment group.

To verify that whether PI3-K (phosphatidylinositide-3′-OH kinase)/Akt (serine-threonine kinase c-Akt, also known as protein kinase B)/GSK3*β* (glycogen synthase kinse 3-*β*) pathway is involved in the protective effect of QP water extract on PC12 cells, LY294002 and SB415286, which are specific inhibitors of PI3-K and GSK3, were used. As shown in Figures 8(a) and 8(b), LY294002 (30 *μ*M) had abolished the protective effect of QP water extract on PC12 cells to a certain level (54.12 ± 1.03%, *P* < 0.001) while SB415286 (30 *μ*M) had shown the protective effects on 6-OHDA-induced death of PC12 cells (72.46 ± 3.86%, *P* < 0.001).

Moreover, the effects of QP water extract on the phophorylayed level of PI3-K/Akt/GSK3*β* were also checked by Western Blot assay. As shown in Figures 8(c) and 8(d), the level of phospho-PI3-K/Akt/GSK3*β* had decreased remarkably after exposure to 500 *μ*M 6-OHDA for 60 min. However, the inhibition of the phosphorylation could be reversed by pretreatment with QP water extract (0.5 mg/mL, 2 h) and the protective effect could be abolished by LY294002 at the same time.

## 4. Discussion

ROS is known as the reactive molecules, including oxygen radicals such as hydroperoxyl and superoxide radical and nonradical derivatives of oxygen such as hydrogen peroxide [13]. Overproduction of such kind of free radical may bring about oxidative damage to biomolecules and eventually lead to chronic diseases [14]. In neurodegenerative diseases, such as Alzheimer's disease (AD) and Parkinson's disease (PD), ROS does play a nonnegligible role [5]. It has been reported that both endogenous and exogenous ROS increases and/or the impairment of antioxidant defense systems could result in enhanced oxidative stress and cell apoptosis [15].

In the present study, both DPPH scavenging activity and total phenolic content were used to evaluate the antioxidant properties of QP water extract against ROS. As shown in the results, QP water extract scavenged the free radicals produced by DPPH solution in a concentration-dependent manner with SC_50_ equal to 0.15 mg/mL and its free radical scavenging ability is relatively lower than those of some common Chinese herbs, for example,* Cratoxylum cochinchinense, Cortex magnoliae officinalis, Psoralea corylifolia *L.*, Curculigo orchioides *Gaertn*., *and* Glycyrrhiza uralensis *Fisch. [5]. In the current study, total phenolic contents were also measured using Folin-Ciocalteu Method [16]. The total phenolic content of QP water extract was found to be 200.78 ± 2.65 mg GAE/g, which is relatively higher than those of some water extracts from* Baccharis genistelloides*,* Physalis alkekengi*,* Taraxacum mongolicun*, and so forth [17].

6-OHDA, a hydroxylated dopamine analogue, can be taken up by the dopamine transporter and causes the release of free radicals that exert toxicity in neuronal tissues [18]. It has been extensively used as an inducer of oxidative stress on neuronal cells. To mimic the disease situation, PC12 cells were exposed to 6-OHDA to induce cell damage. The current results demonstrated that PC12 cells pretreated with QP water extract (0.5 mg/mL, 2 h) could significantly improve the cell viability (Figure 3(b)). It is found that the protective effect of QP water extract on PC12 cells may result from reducing intracellular ROS caused by 6-OHDA (Figure 4). The antiapoptotic effect of QP water extract was further confirmed by morphological observations (Figure 5). With the help of Hoechst 33342 staining, it was found that cells pretreated with QP water extract (0.5 mg/mL, 2 h) before challenging with 6-OHDA (500 *μ*M, 6 h) showed less symptoms of apoptosis as compared with those exposed to 6-OHDA alone.

Mitochondrion is one of the main targets of ROS and its dysfunction in the mitophagy pathway can lead to several neurodegenerative disorders [19, 20]. Using CCCP, which usually acts as an ionophore, could quickly and universally dissipate the proton gradient of the mitochondrial inner membrane [20]. It was used in this study to confirm that JC-1 response is sensitive to changes in membrane potential. QP water extract could significantly reverse the depolarization level of mitochondrial membrane caused by 6-OHDA (Figures 6(a) and 6(b)).

It has been verified in multiple studies that apoptosis is among the main causes that lead to oxidative stress-induced neurodegenerative disease [10]. Among the signal transduction pathways leading to apoptosis, ERKs are one of the well-characterized pathways [21–24]. Moreover, it had received much more attention these years because of its high frequency of KRAS and BRAF mutations identified in certain human cancers and the critical role this pathway plays in promoting cell survival [25]. Along the cascade, the members of the each echelon, from the Raf family to p90 ribosomal S6 kinase (RSK), are activated one by one [26], among which the phosphorylation of mitogen-activated protein kinases (MAPKs) plays a dominant role [27]. And the phosphorylated level of ERK 1/2 and its upstream signal MEK 1/2 had been proved earlier that it could promote 6-OHDA induced cell damages in PC12 cells [16, 28]. It is shown with the treatment of PD98059 that ERK pathway was involved in 6-OHDA-induced apoptosis of PC12 cells. What is more, the level of phosphorylation of ERK 1/2 and MEK 1/2 is attenuated with 2 h pretreatment of QP water extract, which indicated that QP water extract did protect the PC12 cells from apoptosis by eliminating the increase in phospho-ERK induced by 6-OHDA.

Apart from MEK/ERK pathway, the PI3-K/Akt/GSK3*β* pathway may be another vital target to improve cells survival [29]. To be specific, PI3-K/Akt are well defined as mediators for cell survival, activated by some peptide factors including the neurotrophins and growth factors [30]. By using of specific PI3-K inhibitor LY294002, the effect of QP water extract was blocked (Figures 8(a)–8(d)), which indicating PI3-K/Akt is involved in the protective effect of QP water extract. On the other hand, GSK3*β*, another regulator related to cell apoptosis, was defined as the downstream kinase of PI3-K/Akt in some of the primary studies [31, 32]. The activity of GSK3*β* is positively regulated by 6-OHDA at tyrosine 216 (Tyr216) but negatively at serine 9 (ser9) by phosphorylation [33]. Through the use of GSK3*β* inhibitor SB415286, we had verified that GSK3*β* is involved in the 6-OHDA-induced PC12 cell damage (Figure 8(b)). Regulated by PI3-K/Akt, the inactivation of downstream GSK3*β* through phosphorylation at ser9 by QP water extract can also explain its protective effect on PC12 cells. As a result, we proposed that PI3-K/Akt/GSK3*β* pathway is involved in this model. Besides, it was demonstrated that the reduction in the level of phosphorylated PI3-K/Akt/GSK3*β* was reversed by pretreatment with QP water extract (0.5 mg/mL, 2 h), which indicated that it could protect PC12 cells from 6-OHDA induced apoptosis by reversing the inhibition of PI3-K/Akt/GSK3*β* pathway.

Although we demonstrated that QP water extract has relatively high neuroprotective capacity using* in vitro* cell model, further studies are still needed to determine QP's neuroprotective effects against intracellular ROS-induced oxidative stress. Upregulation of antioxidant enzymes such as SOD, GPx, and CAT is one of the major defense systems to handle oxidative stress [34]. Targeting these enzymes could be one of the underlying mechanisms of QP water extract's effects. Further study on this area may provide more insights on the mechanisms of QP.

Due to the high level of oxygen consumption of brain, the nervous system is usually more vulnerable to the damage of homeostasis between the production of ROS and defense mechanisms [35]. Normal dopaminergic transmission plays an important role in regulating neuronal functions and previous studies had shown that there may be a correlation ship between the oxidative stress, as well as ROS induced apoptosis, and dopaminergic alteration [36]. On the other hand, in PD patients, selective vulnerability of dopaminergic neuron is found and dopamine (DA) autooxidation together with its metabolism by monoamine oxidase B would produce 6-OHDA as well as dopamine quinones, which could generate ROS [37]. In the present study, by using of 6-OHDA and PC12 cells, the main pathology of PD was mimicked* in vitro*, and QP water extract was verified to have the capacity of scavenging ROS produced by 6-OHDA, which could provide evidence of its potential in developing innovated agent for treatment of PD.

Since there was no study demonstrating that QP water extract has neuroprotective capacity, it is still unknown that whether it can cross brain barrier. We had found that there are four main constituents in QP water extract through HPLC, including exculin hydrate, fraxin, esculetin, and flaxetin. According to their chemical property, exculin and fraxin have high polarity while esculetin and flaxetin have relatively low polarity, which make them easier to cross the brain barrier. In particular, there are previous studies showing that esculetin and flaxetin could prevent the neurotoxicity caused by agents other than 6-OHDA and they were reported to be easy to cross blood-brain barrier [38, 39]. However, further studies using* in vivo* animal model or* in vitro* tissue culture are needed to clarify whether it QP water extract, a TCM with complex constituents, can cross the brain barrier as whole.

## 5. Conclusion

In summary, QP water extract was demonstrated to have strong antioxidant property in DPPH assay with SC_50_ = 0.15 mg/mL. Total phenolic content of QP water extract was found to be 200.78 ± 2.65 mg GAE/g. Additionally, QP water extract (0.5 mg/mL) could remarkably increase (*P* < 0.001) the viability of PC12 cells treated with 6-OHDA as compared with the vehicle and reverse-increase intracellular ROS level at the same time. The antiapoptotic effects of QP water extract was shown in Hoechst staining assay and flow cytometry analysis. The protective effect of QP water extract was found to be via inhibiting MEK/ERK pathway and reversing PI3-K/Akt/GSK3*β* pathway. The current results suggest that QP might be a potential candidate for preventing the development of neurodegenerative diseases, such as PD.

## Abbreviations

PD: Parkinson's disease
DMSO: Dimethyl sulfoxide
DPPH: 1,1-Diphenyl-2-picrylhydrazyl
ROS: Reactive oxygen species
SR%: Free radical scavenging capacity
SC_50_: Concentration of sample that contributes 50% of scavenging capacity
QP: Cortex Fraxini (Qingpi)
6-OHDA: 6-Hydroxydopamine
PC12 cells: Rat pheochromocytoma cells
TCM: Traditional chinese medicine
MTT: 3-(4,5-Dimethylthiazol-2yl)-2,5-diphenyltetrazolium brmide.
